# Thalamic white matter macrostructure and subnuclei volumes in Parkinson’s disease depression

**DOI:** 10.1038/s41531-021-00270-y

**Published:** 2022-01-10

**Authors:** R. Bhome, A. Zarkali, G. E. C. Thomas, J. E. Iglesias, J. H. Cole, R. S. Weil

**Affiliations:** 1 Dementia Research Centre, University College London, London, UK; 2 Centre for Medical Image Computing, Department of Computer Science, University College London, London, UK; 3 Martinos Center for Biomedical Imaging, Massachusetts General Hospital and Harvard Medical School, Cambridge, USA; 4 Computer Science and Artificial Intelligence Laboratory, Massachusetts Institute of Technology, Cambridge, USA; 5 Wellcome Centre for Human Neuroimaging, University College London, London, UK; 6 Movement Disorders Consortium, National Hospital for Neurology and Neurosurgery, London, UK

**Keywords:** Parkinson's disease, Diagnostic markers

## Abstract

Depression is a common non-motor feature of Parkinson’s disease (PD) which confers significant morbidity and is challenging to treat. The thalamus is a key component in the basal ganglia-thalamocortical network critical to the pathogenesis of PD and depression but the precise thalamic subnuclei involved in PD depression have not been identified. We performed structural and diffusion-weighted imaging (DWI) on 76 participants with PD to evaluate the relationship between PD depression and grey and white matter thalamic subnuclear changes. We used a thalamic segmentation method to divide the thalamus into its 50 constituent subnuclei (25 each hemisphere). Fixel-based analysis was used to calculate mean fibre cross-section (FC) for white matter tracts connected to each subnucleus. We assessed volume and FC at baseline and 14–20 months follow-up. A generalised linear mixed model was used to evaluate the relationship between depression, subnuclei volume and mean FC for each thalamic subnucleus. We found that depression scores in PD were associated with lower right pulvinar anterior (PuA) subnucleus volume. Antidepressant use was associated with higher right PuA volume suggesting a possible protective effect of treatment. After follow-up, depression scores were associated with reduced white matter tract macrostructure across almost all tracts connected to thalamic subnuclei. In conclusion, our work implicates the right PuA as a relevant neural structure in PD depression and future work should evaluate its potential as a therapeutic target for PD depression.

## Introduction

Depression is a common neuropsychiatric feature of Parkinson’s disease (PD) with a prevalence of around 35%^1^. It is also the key health-related determinant of poor quality of life in PD^2^. There is converging evidence to suggest that depression in PD arises primarily from the underlying neurobiology of the disorder rather than due to a psychological response to functional impairment. For example, depressive symptoms often arise in the prodromal stage prior to hallmark PD motor symptoms and genetic mutations linked to PD confer a risk of affective psychopathology^3,4^.

A clear understanding of the neural correlates of PD depression has remained elusive, with functional, structural, and nuclear imaging studies yielding broad findings^5,6^. The frontotemporal regions, thalamus, amygdala, cerebellar white matter, hippocampus, nucleus accumbens, globus pallidus, anterior cingulate cortex and insula have all been implicated as potentially relevant brain regions and all three major monoaminergic systems are likely to be involved in the underlying pathophysiology^5,6^.

One brain structure that warrants further investigation is the thalamus. It occupies a pivotal position in the basal ganglia-thalamocortical networks that are affected both in the pathogenesis of PD and mood disorders^7,8^. In PD depression specifically, functional magnetic resonance imaging (fMRI) studies show increased activation in the left mediodorsal thalamus^9^, as well as increased connectivity between the left amygdala and bilateral mediodorsal thalami in depressed compared to non-depressed PD patients^10^. Although white matter microstructural changes^11^ and noradrenergic denervation have been identified in the mediodorsal regions of the thalamus^12^, those studies were not able to examine changes within individual thalamic subnuclei, limiting anatomical precision. The benefit of greater precision in defining neuroanatomical regions affected in PD depression is the potential of these locations as targets for therapeutic intervention, given their pivotal role in modulating brain circuits.

Previous work has demonstrated that antidepressants may modulate thalamic circuitry involving the pulvinar and mediodorsal subnuclei in depression in general^13–15^. Structural changes can be found in regions with altered functional connectivity following antidepressant treatment^16^ and antidepressants may reduce grey matter shrinkage of affected regions through neuroprotective, neurogenesis and neuro-modulatory effects^17–19^. Indeed, alterations in grey matter volume by antidepressants have been demonstrated in the broader depression literature^20^ but a region of interest volumetric analysis, which did not include the thalamus, did not find any difference between antidepressant treated PD patients and non-depressed PD patients^21^. Therefore, an aim of this study was to investigate whether antidepressant use modifies thalamic changes caused by PD depression.

Until recently, analysing thalamic subnuclei morphology and the specific neural connections of individual nuclei has proven problematic as this has required manual segmentation of thalamic subnuclei which can be laborious as well as error-prone. Recently, however, a novel technique has been developed, that uses a Bayesian framework based on probabilistic atlases (derived from ex-vivo histology) and unsupervised appearance modelling to robustly segment these complex deep nuclei^22^.

Here, we investigated how levels of depression amongst PD patients influence thalamic subnucleus volume and white matter tracts connected to thalamic subnuclei. We hypothesised that depression would be associated with lower volume and altered white matter structure within thalamic subnuclei. We further examined the effects of antidepressant use on the pulvinar and mediodorsal subnuclei in PD, hypothesising that there would be volume and white matter structure changes although the expected direction was uncertain based on previous work^14,15^.

## Results

### Demographic and clinical characteristics

102 participants were included; 76 with PD and 26 HC, as described in previous work from our group^23,24^ (where slight differences in included numbers are seen, these relate to exclusions based on quality control of images, relevant to each study question). As described previously, age, gender and years of education did not differ between groups. At baseline, the PD group had significantly higher scores than HC on Hospital Anxiety and Depression Scale (HADS) anxiety and HADS depression, suggesting increased affective psychopathology (see Table 1), as would be expected in PD. On cognitive testing, the PD group scored significantly lower than HC on Montreal Cognitive Assessment (MoCA) but the average score for both groups was within the normal range (≥26). Scores on specific tests of language, visuospatial ability, executive functioning and memory, both immediate and delayed, did not differ between groups. The PD group had significantly higher scores than HC on disease-specific measures including total Movement Disorders Society Unified Parkinson’s Disease Rating Scale (MDS-UPDRS), MDS-UPDRS motor score, and REM sleep scores, as expected but there was no significant difference in hallucinations score (see Table 1).

**Table 1:** Demographics, cognitive and disease specific measures of participants with PD and HC.

Attribute	PD (*n* = 76)	HC (*n* = 26)	Statistic
*Demographics*			
Age, year	64.58 (7.98)	67.42 (8.02)	*t* = −1.55, *p* = 0.12
Male, *n* (%)	42 (55)	12 (46)	*χ*^2^ = 0.33, *p* = 0.56
Education, year	17.26 (2.85)	17.85 (2.16)	*U* = 884.0, *p* = 0.42
Handedness, (*n*, Right) (%)	61 (80)	24 (92)	*χ*^2^ = 1.25, *p* = 0.26
*Mood (HADS)*			
**Depression score**	**3.95 (3.17)**	**1.15 (1.49)**	***U*** = **1591.5,** ***p*** = **2.79** **×** **10**^**−6**^
**Anxiety score**	**5.70 (3.77)**	**3.54 (3.43)**	***U*** = **1333.5,** ***p*** = **0.008**
*Cognitive testing*			
MMSE	29.01 (1.18)	29.19 (0.88)	*U* = 953.0, *p* = 0.78
**MoCA**	**27.95 (2.20)**	**29.00 (1.14)**	***U*** = **717.0,** ***p*** = **0.03**
Stroop time^a^	62.83 (21.80)	56.22 (14.01)	*U* = 1144.0, *p* = 0.19
Digit Span (forwards)^b^	9.25 (2.00)	9.33 (2.05)	*U* = 348.5, *p* = 0.94
Digit Span (backwards)^b^	7.34 (2.19)	6.92 (2.33)	*t* = 0.59, *p* = 0.55
Log memory delayed^b^	13.25 (4.48)	12.75 (3.32)	*t* = 0.36, *p* = 0.72
Log memory immediate^b^	15.37 (4.44)	15.17 (3.53)	*t* = 0.15, *p* = 0.88
Word recall	24.17 (1.10)	24.46 (1.01)	*U* = 810.5, *p* = 0.13
Fluency letter	17.04 (5.17)	17.81 (4.95)	*U* = 910.0, *p* = 0.55
Fluency categorical	21.72 (5.68)	21.88 (4.66)	*U* = 955.0, *p* = 0.80
GNT	24.12 (2.58)	23.62 (4.15)	*U* = 937.5, *p* = 0.70
HVOT	24.83 (2.96)	25.94 (2.10)	*U* = 769.0, *p* = 0.09
JLO^a^	24.83 (3.93)	26.0 (3.29)	*t* = −1.35, *p* = 0.18
*Disease-specific measures*			
Disease duration, year	4.12 (2.48)		
LEDD	427.85 (219.66)		
Affected side, right (%)	36 (47)		
left (%)	11 (14)		
Bilateral (%)	29 (38)		
**MDS-UPDRS**	**45.87 (21.01)**	**9.54 (5.71)**	***U*** = **1954.5,** ***p*** = **1.17** **×** **10**^**−13**^
**MDS-UPDRS Motor Score**	**23.38 (12.33)**	**5.96 (4.51)**	***U*** = **1864.5,** ***p*=1.69** **×** **10**^**−11**^
**RBDSQ**	**4.16 (2.25)**	**1.96 (1.51)**	***U*** = **1535.0,** ***p*** = **2.24** **×** **10**^**−5**^
UM-PDHQ	0.68 (1.85)	0.04 (0.19)	*U* = 1111.0, *p* = 0.10

All data shown are mean (SD) except sex, handedness and affected side. Significant differences are highlighted in bold text.

*HADS* Hospital Anxiety and Depression Scale, *MMSE* mini-mental state examination, *MoCA* Montreal cognitive assessment, *Stroop time* time for completion of both word and colour tasks, *Log memory* logical memory, *GNT* Graded Naming Test, *HVOT* Hooper visual organisation test, *JLO* judgement of line orientation, *LEDD* total levodopa equivalent dose, *MDS-UPDRS* Movement Disorders Society Unified Parkinson’s Disease Rating Scale, *RBDSQ* REM Sleep Behaviour Disorder Screening Questionnaire, *UM-PDHQ* University of Miami Hallucinations Questionnaire.

^a^PD (*n* = 75).

^b^PD (*n* = 59).

### Greater severity in disease measures associated with depression

In PD, at baseline, increased HADS depression score was significantly associated (post FDR correction) with higher anxiety scores (measured using HADS) and total MDS-UPDRS scores which is an overall measure of Parkinson’s disease severity (Table 2). HADS depression scores did not change significantly between baseline and follow-up in PD participants (baseline, mean = 3.95 (SD = 3.17); follow up, mean = 4.24 (SD = 3.70), *U* = 2829.5, *p* = 0.83), supporting our decision to use an average HADS depression score across time points in subsequent analyses.

**Table 2:** Association of HADS depression score with baseline measure and follow-up scores in anxiety, cognitive and disease-specific measures.

Attribute	Baseline mean (SD)	Beta	*p* value^a^	*q* value^b^	Longitudinal change mean (SD)	Beta	*p* value^a^	*q* value^b^
*Mood (HADS)*								
Anxiety score (*n* = 76)^c^	**5.70 (3.77)**	**0.70**	**<0.001**	**0.01**	**−0.43 (2.98)**	**0.70**	**<0.001**	**0.006**
*Cognitive testing*								
MMSE (*n* = 76)^c^	29.01 (1.18)	−0.11	0.01	0.06	**−0.07 (1.51)**	**−0.12**	**<0.001**	**0.006**
MoCA (*n* = 76)^c^	27.95 (2.20)	−0.08	0.33	0.48	−0.20 (2.18)	−0.10	0.21	0.32
Stroop-time (*n* = 75)^d^	62.83 (21.80)	1.34	0.07	0.19	−0.74 (23.40)	1.43	0.03	0.08
Digit Span (forwards) (*n* = 59)	9.25 (2.00)	−0.03	0.74	0.78	0.58 (1.76)	−0.03	0.69	0.73
Digit Span (backwards) (*n* = 59)	7.34 (2.19)	−0.05	0.57	0.68	0.47 (1.87)	−0.10	0.30	0.40
Fluency letter (*n* = 76)^c^	13.25 (4.48)	0.05	0.81	0.81	−0.44 (4.24)	−0.09	0.63	0.71
Fluency category (*n* = 76)^c^	15.37 (4.44)	−0.08	0.71	0.78	−1.57 (5.13)	−0.28	0.15	0.25
Log Memory Delayed (*n* = 59)	24.17 (1.10)	−0.27	0.15	0.36	−1.78 (3.78)	−0.16	0.31	0.40
Log Memory Immediate (*n* = 59)	17.04 (5.17)	−0.14	0.46	0.58	−2.79 (4.67)	−0.02	0.89	0.89
Word recall (*n* = 76)^c^	21.72 (5.68)	−0.05	0.26	0.45	−0.17 (1.20)	−0.02	0.60	0.71
GNT (*n* = 76)^c^	24.12 (2.58)	−0.18	0.05	0.19	**0.51 (1.78)**	**−0.28**	**0.006**	**0.02**
HVOT (*n* = 76)^c^	24.83 (2.96)	−0.23	0.02	0.10	0.13 (2.29)	−0.20	0.06	0.11
JLO (*n* = 75)^e^	24.83 (3.93)	−0.12	0.39	0.53	**−0.15 (3.24)**	**−0.38**	**0.004**	**0.02**
*Disease-specific measures*								
MDS-UPDRS (*n* = 76)	**45.87 (21.01)**	**3.07**	**<0.001**	**0.01**	**−0.79 (17.21)**	**2.98**	**<0.001**	**0.006**
MDS-UPDRS Motor Score (*n* = 76)	23.38 (12.33)	0.78	0.07	0.19	**−0.84 (12.40)**	**0.78**	**0.01**	**0.03**
RBDSQ (*n* = 76)	4.16 (2.25)	0.10	0.20	0.42	0.45 (2.20)	0.18	0.04	0.08
UM-PDHQ (*n* = 76)	0.68 (1.85)	0.08	0.23	0.44	0.05 (1.68)	0.13	0.04	0.08

In bold results showing FDR-corrected statistically significant associations.

*HADS* Hospital Anxiety and Depression Scale, *MMSE* Mini-Mental State Examination, *MoCA* Montreal Cognitive Assessment, *Stroop time* time for completion of both word and colour tasks, *log memory* logical memory, *GNT* Graded Naming Test, *HVOT* Hooper Visual Organisation Test, *JLO* judgement of line orientation, *LEDD* total levodopa equivalent dose, *MDS-UPDRS* Movement Disorders Society Unified Parkinson’s Disease Rating Scale, *RBDSQ* REM Sleep Behaviour Disorder Screening Questionnaire, *UM-PDHQ* University of Miami Hallucinations Questionnaire

^a^*P* values were analysed by a GLMM. At baseline, for anxiety score there were no co-variates, for cognitive measures, age was a co-variate and for disease-specific measures, LEDD and disease duration were co-variates. For follow-up scores, the time between visits was an additional co-variate. The participant was a random effect in these models.

^b^*q* values were calculated following FDR correction using the Benjamini–Hochberg method.

^c^*n* = 75 for longitudinal change scores.

^d^*n* = 74 for longitudinal change scores.

^e^*n* = 73 for longitudinal change scores.

Average HADS depression scores were associated with poorer performance at follow-up in Mini-Mental State Examination (MMSE), Graded Naming Test (GNT) and Judgement of Line Orientation (JLO) (Table 2). In addition, higher depression scores were significantly related to worse scores on the total MDS-UPDRS and MDS-UPDRS motor score. However, an association between depression scores and REM sleep disorder, measured by REM Sleep Behaviour Disorder Screening Questionnaire (RBDSQ), and hallucination severity, measured by University of Miami Hallucinations Questionnaire (UM-PDHQ), did not survive false discovery rate (FDR) correction (Table 2).

### Robustness of thalamic subnuclei segmentation

The posterior probability distributions utilised for thalamic segmentation were consistent between subjects and hemispheres but varied between subnuclei. Subnuclei with lower distributions were therefore consistently less reliably segmented across subjects, whereas those with higher distributions were consistently more reliably identified. The complete set of mean posterior probability distributions can be seen in Supplementary Fig. 1.

### Thalamic subnuclei volumes and depression

Depression severity was not significantly associated with baseline volumes of any of the thalamic subnuclei (Supplementary Table 1). Depression scores were associated with lower right PuA volume after follow-up but this did not survive FDR correction for multiple comparisons (*β* = −1.32 (SE = 0.52), *p* = 0.01, *q* = 0.50)) and with lower right pulvinar medial (PuM) volume after follow-up although this was above statistical significance levels (*β* = −5.46 (SE = 2.79), *p* = 0.05, *q* = 0.72) (Table 3).

**Table 3:** Association of HADS depression score with thalamic subnuclei volumes.

Nuclei group	Left thalamus					Right thalamus				
	Baseline volume (mm^3^)^a^	Longitudinal change (mm^3^)^a^	Beta	*p* value^b^	*q* value^c^	Baseline volume (mm^3^)^a^	Longitudinal change (mm^3^)^a^	Beta	*p* value^b^	*q* value^c^
*Anterior*										
AV	126.76 (15.77)	−0.69 (7.74)	0.21	0.66	0.87	141.07 (18.03)	0.21 (8.29)	0.15	0.79	0.95
*Lateral*										
LD	27.33 (7.29)	−0.01 (2.69)	0.03	0.90	0.97	26.20 (5.76)	0.17 (3.30)	0.30	0.16	0.80
LP	122.09 (15.51)	−0.24 (8.89)	−0.06	0.88	0.97	109.67 (13.66)	0.54 (8.23)	0.44	0.26	0.85
*Ventral*										
VA	381.10 (46.16)	−2.02 (23.49)	0.54	0.59	0.87	392.75 (46.23)	−1.50 (21.64)	−0.90	0.42	0.85
VAmc	27.91 (3.56)	−0.35 (2.18)	0.009	0.91	0.97	29.35 (3.68)	−0.11 (2.00)	−0.05	0.54	0.87
VLa	594.93 (69.07)	−5.39 (32.26)	−0.70	0.66	0.87	616.26 (69.72)	−0.94 (29.77)	−1.28	0.43	0.85
VLp	772.62 (84.59)	−7.12 (40.77)	−1.86	0.36	0.85	793.23 (86.38)	−2.52 (37.12)	−1.35	0.52	0.87
VPL	800.84 (95.13)	−13.97 (50.60)	−1.82	0.44	0.85	807.80 (99.60)	−5.27 (41.80)	−1.19	0.64	0.87
VM	18.94 (2.35)	−0.30 (1.27)	−0.02	0.75	0.95	19.62 (2.65)	−0.17 (1.21)	–0.02	0.82	0.95
*Intralaminar*										
CeM	58.50 (8.40)	−0.45 (5.35)	−0.18	0.50	0.87	62.14 (8.92)	0.09 (4.71)	−0.23	0.42	0.85
CL	36.53 (6.40)	0.13 (3.71)	0.25	0.26	0.85	37.08 (5.02)	0.18 (3.89)	0.18	0.28	0.85
Pc	3.06 (0.48)	−0.05 (0.29)	0.008	0.56	0.87	3.15 (0.51)	−0.09 (0.28)	−0.02	0.07	0.72
CM	241. 14 (28.54)	−2.26 (14.77)	0.37	0.61	0.87	241.29 (27.40)	−1.79 (14.05)	−0.13	0.85	0.97
Pf	53.49 (6.58)	−0.32 (3.84)	0.27	0.13	0.72	55.24 (6.05)	0.01 (3.73)	0.14	0.37	0.85
*Medial*										
Pt	6.80 (0.79)	−0.003 (0.41)	0.000	0.99	0.99	6.63 (0.69)	0.01 (0.38)	0.001	0.97	0.99
MV-re	9.63 (1.85)	−0.05 (1.41)	−0.01	0.81	0.95	9.81 (2.04)	−0.02 (1.54)	−0.06	0.40	0.85
MDm	624.67 (82.33)	−9.78 (43.98)	−3.68	0.08	0.72	649.59 (82.40)	−12.64 (56.65)	−2.74	0.26	0.85
MDl	245.00 (27.60)	−0.93 (15.29)	−1.09	0.12	0.72	260.49 (25.22)	−0.81 (14.00)	−0.97	0.13	0.72
*Posterior*										
LGN	158.63 (27.79)	−2.81 (14.03)	−1.30	0.08	0.72	170.08 (27.59)	0.43 (13.89)	−0.31	0.66	0.87
MGN	114.02 (18.71)	−2.12 (11.30)	−0.50	0.34	0.85	114.23 (18.05)	−1.43 (9.89)	−0.69	0.12	0.72
L-SG	24.69 (5.15)	0.64 (3.55)	−0.04	0.77	0.95	23.78 (5.18)	0.22 (3.50)	−0.09	0.53	0.87
PuA	194.63 (21.69)	-4.16 (10.70)	-0.50	0.33	0.85	**214.48 (23.29)**	**−2.02 (14.00)**	**−1.32**	**0.01**	**0.50**
PuM	939.30 (105.19)	−12.68 (48.58)	−3.36	0.25	0.85	979.63 (106.99)	−3.65 (57.17)	−5.46	0.05	0.72
PuL	183.23 (30.12)	−0.88 (15.15)	−0.04	0.97	0.99	205.28 (31.97)	3.64 (15.30)	−0.97	0.30	0.85
PuI	171.55 (23.62)	−2.11 (16.32)	−0.33	0.62	0.87	189.12 (23.49)	−1.05 (18.81)	−0.58	0.38	0.85

The result in bold represents an association that did not survive FDR correction.

Anterior nuclei: *AV* anteroventral; Lateral nuclei: *LD* laterodorsal, *LP* lateral posterior, Ventral nuclei: *VA* ventral anterior, *VAmc* ventral anterior magnocellular, *VLa* ventral lateral anterior, *VLp* ventral lateral posterior, *VPL* ventral posterolateral, *VM* ventromedial; Intralaminar nuclei: *CeM* central medial, *CL* central lateral, *Pc* paracentral, *CM* centromedian, *Pf* parafascicular; Medial nuclei: *Pt* paratenial, *MV-re* reuniens (medial ventral), *MDm* mediodorsal medial magnocellular, *MDl* mediodorsal lateral parvocellular; Posterior nuclei: *LGN* lateral geniculate, *MGN* medical geniculate, *L-SG* limitans (suprageniculate), *PuA* pulvinar anterior, *PuM* pulvinar medial, *PuL* pulvinar lateral, *PuI* pulvinar inferior.

^a^For each nucleus, baseline volume and longitudinal change are presented as mean (SD).

^b^*p* values were analysed by a GLMM corrected by age, gender total intracranial volume, time between visits with participants as a random effect.

^c^*q* values were calculated following FDR correction using the Benjamini–Hochberg method.

Post-hoc, based on the observation that depression in Parkinson’s disease is often associated with poorer cognition^25^, we additionally covaried for MOCA scores. We found that the association between depression and lower right PuA remained, suggesting that our finding is likely to be specific to depression, and not confounded by poorer global cognition in these patients (Supplementary Table 2).

Given the significant association between HADS depression and HADS anxiety scores, we evaluated the relationship of average HADS anxiety scores with thalamic subnuclei volumes using the same GLMM as that used to evaluate the association of depression with thalamic subnuclei volumes (see the “Methods” section). There were no significant associations between HADS anxiety scores and any subnuclei including the right PuA (*β* = −0.37 (SE = 0.52), *p* = 0.47) and right PuM (*β* = −1.81 (SE = 2.73), *p* = 0.51), at follow-up (Supplementary Table 3).

### Effects of antidepressant use on thalamic sub-nuclei

We examined the effects of antidepressant use. In our sample, nine participants with PD were taking antidepressants and did not differ in clinical measures from the participants with PD who were not taking antidepressants (n = 67). In particular, there were no significant differences in mean HADS depression scores between those taking (*n* = 9, 5.39 (SD = 4.38)) and not taking antidepressants (*n* = 67, 3.92 (SD = 2.99), *U* = 359.0, *p* = 0.36) (Supplementary Table 4).

**Fig. 1:**
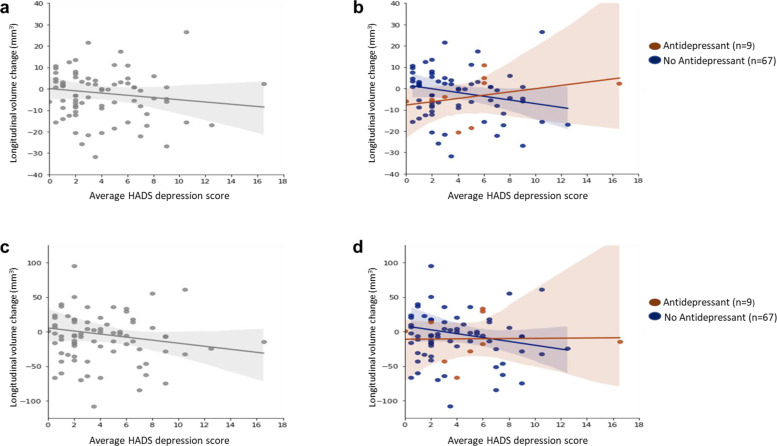
Relationship between HADS depression score and longitudinal change in pulvinar anterior and pulvinar medial volumes. The top panels show scatter plots for longitudinal change in pulvinar anterior (PuA) volume in relation to average HADS depression score for all PD participants (**a**) and also separately for PD participants taking (brown) or not taking (blue) antidepressants (**b**). The corresponding scatter plots for pulvinar medial (PuM) volume change in relation to average HADS depression is shown in the bottom panels for all PD participants (**c**) and separately for participants taking (brown) or not taking (blue) antidepressants (**d**). Overall, higher HADS depression scores were associated with volume decreases in the right PuA (*r* = −0.12, *P* = 0.30) (**a**) and right PuM (*r* = −0.12, *P* = 0.28) (**c**). However, when participants were separated into those taking or not taking antidepressants, there was a positive correlation between HADS depression scores and longitudinal volume change in the right PuA for those taking antidepressants (*r* = 0.34, *P* = 0.37) but a negative correlation for those not taking antidepressants (*r* = −0.18, *P* = 0.14) (**b**). There was a similar pattern for the right PuM; Antidepressant group (*r* = 0.02, *P* = 0.96); No antidepressant group (*r* = −0.14, *P* = 0.26) (**d**).

Adding antidepressant use as a covariate in the analysis strengthened the regression model which had subnuclei volume as the dependent variable, average HADS depression score as the independent variable and time between visits, age, intracranial volume and gender as covariates (with antidepressant use, AIC = 1245.6; without antidepressant use, AIC = 1249.2, *p* = 0.02). In this model, the association between depression scores and volume remained significant in the right pulvinar anterior (PuA) (*β* = −1.57 (SE = 0.52), *p* = 0.003) and became significant for the right PuM (*β* = −6.40 (SE = 2.83), *p* = 0.02) at follow-up. Antidepressant use was significantly associated with higher right PuA volume at follow-up (*β* = 12.27 (SE = 5.34), *p* = 0.02). We further found that in these subnuclei, for a higher depression score, patients taking antidepressants showed less volume loss than those not taking antidepressants but this relationship did not reach statistical significance (Fig. 1).

### Fibre cross-section changes associated with depression

At baseline, there were no significant relationships between depression scores and FC (Supplementary Table 5). After follow-up, we found a significant association between HADS depression scores and FC for tracts connected to all thalamic subnuclei apart from the left PuA, left paratenial (Pt) and right Pt (Table 4). This relationship was of reduced FC with higher depression scores for all tracts apart from the ventromedial (VM) and paracentral (Pc) nuclei bilaterally, which showed the opposite relationship, with increased FC with higher depression severity. All significant associations survived correction for multiple comparisons.

**Table 4:** Association of average HADS depression score with follow up thalamic subnuclei FC scores.

Nuclei group	Left thalamus					Right thalamus				
	Baseline FC^a^	Longitudinal change^a^	beta	*p* value^b^	*q* value^c^	Baseline FC^a^	Longitudinal change^a^	Beta	*p* value^b^	*q* value^c^
*Anterior*										
AV	**0.071**	**−0.197**	**−0.011**	**0.018**	**0.026**	**0.078**	**−0.199**	**−0.012**	**0.014**	**0.026**
*Lateral*										
LD	**0.085**	**−0.198**	**−0.012**	**0.015**	**0.026**	**0.083**	**−0.199**	**−0.012**	**0.013**	**0.026**
LP	**0.016**	**−0.168**	**−0.012**	**0.007**	**0.026**	**0.075**	**−0.180**	**−0.012**	**0.010**	**0.026**
*Ventral*										
VA	**0.070**	**−0.202**	**−0.011**	**0.026**	**0.029**	**0.071**	**−0.205**	**−0.011**	**0.017**	**0.026**
VAmc	**0.067**	**−0.196**	**−0.011**	**0.018**	**0.026**	**0.072**	**−0.206**	**−0.011**	**0.017**	**0.026**
VLa	**0.067**	**−0.202**	**−0.011**	**0.028**	**0.026**	**0.066**	**−0.206**	**−0.011**	**0.020**	**0.026**
VLp	**0.066**	**−0.202**	**−0.010**	**0.030**	**0.033**	**0.067**	**−0.205**	**−0.011**	**0.021**	**0.026**
VPL	**0.069**	**−0.200**	**−0.011**	**0.025**	**0.028**	**0.065**	**−0.202**	**−0.011**	**0.021**	**0.026**
VM	**−0.300**	**0.833**	**0.043**	**0.031**	**0.033**	**−0.287**	**0.834**	**0.044**	**0.020**	**0.026**
*Intralaminar*										
CeM	**0.065**	**−0.194**	−**0.011**	**0.020**	**0.026**	**0.068**	**−0.197**	**−0.011**	**0.019**	**0.026**
CL	**0.083**	**-0.194**	**−0.011**	**0.014**	**0.026**	**0.085**	**−0.205**	**−0.014**	**0.017**	**0.026**
Pc	**−0.217**	**0.585**	**0.034**	**0.017**	**0.026**	**−0.227**	**0.605**	**0.036**	**0.014**	**0.026**
CM	**0.070**	**−0.198**	**−0.011**	**0.019**	**0.026**	**0.073**	**−0.202**	**−0.011**	**0.017**	**0.026**
Pf	**0.062**	**−0.190**	**−0.011**	**0.018**	**0.026**	**0.069**	**−0.196**	**−0.011**	**0.018**	**0.026**
*Medial*										
Pt	−0.217	−0.217	0.009	0.080	0.083	−0.227	−0.227	0.009	0.120	0.12
MV-re	**0.069**	**−0.196**	**−0.011**	**0.017**	**0.026**	**0.074**	**−0.202**	**−0.011**	**0.016**	**0.026**
MDm	**0.039**	**−0.168**	**−0.011**	**0.010**	**0.026**	**0.039**	**−0.177**	**−0.010**	**0.020**	**0.026**
MDl	**0.016**	**−0.161**	**−0.011**	**0.012**	**0.026**	**−0.007**	**−0.110**	**−0.010**	**0.007**	**0.026**
*Posterior*										
LGN	**0.081**	**−0.207**	**−0.011**	**0.022**	**0.026**	**0.074**	**−0.199**	**−0.012**	**0.013**	**0.026**
MGN	**0.080**	**−0.200**	**−0.011**	**0.018**	**0.026**	**0.077**	**−0.199**	**−0.012**	**0.015**	**0.026**
L-SG	**0.069**	**−0.183**	**−0.011**	**0.014**	**0.026**	**0.090**	**−0.195**	**−0.012**	**0.010**	**0.026**
PuA	−0.016	−0.160	−0.010	0.097	0.099	**0.047**	**−0.169**	**−0.012**	**0.007**	**0.026**
PuM	**0.084**	**−0.201**	**−0.011**	**0.017**	**0.026**	**0.080**	**−0.198**	**−0.012**	**0.013**	**0.026**
PuL	**0.076**	**−0.204**	**−0.011**	**0.025**	**0.028**	**0.072**	**−0.205**	**−0.011**	**0.018**	**0.026**
PuI	**0.083**	**−0.205**	**−0.012**	**0.017**	**0.026**	**0.081**	**−0.204**	**−0.012**	**0.014**	**0.026**

In bold results showing FDR-corrected statistically significant associations.

Anterior nuclei: (AV = anteroventral);Lateral nuclei: (LD = laterodorsal, LP = lateral posterior);Ventral nuclei: (VA = ventral anterior, Vamc = ventral anterior magnocellular, VLa = ventral lateral anterior, VLp = ventral lateral posterior, VPL = ventral posterolateral, VM = ventromedial; Intralaminar nuclei: (CeM = central medial, CL = central lateral, Pc = paracentral, CM = centromedian Pf = parafascicular); Medial nuclei: (Pt = paratenial, MV-re = reuniens (medial ventral), MDm = mediodorsal medial magnocellular, MDl = mediodorsal lateral parvocellular); Posterior nuclei: (LGN = lateral geniculate, MGN = medical geniculate, L-SG = limitans (suprageniculate), PuA = pulvinar anterior, PuM = pulvinar medial, PuL = pulvinar lateral, PuI = pulvinar inferior).

^a^For each nucleus, baseline FC and longitudinal change are presented as mean (SD).

^b^*p* values were analysed by a GLMM corrected by age, gender total intracranial volume, time between visits with participant as a random effect.

^c^*q* values were calculated following FDR correction using the Benjamini–Hochberg method.

## Discussion

We examined the effects of Parkinson’s depression on thalamic subnuclei employing an automated MRI segmentation method based on a probabilistic atlas derived from histology. This allowed us to analyse individual thalamic subnuclei volumes and the white matter connections of these subnuclei. We found that lower volume in the right pulvinar, specifically the right PuA subnucleus, is associated with depression scores and that antidepressant use was related to higher right PuA volume. Additionally, we found that depression scores were associated with reduced FC across almost all thalamic subnuclei at follow-up. This suggests that depression in PD is associated with widespread loss of white matter macrostructural integrity of fibres projecting to and from the thalamus. Notably, we also found that depression in Parkinson’s disease was associated with worse total MDS-UPDRS scores, motor symptoms, anxiety, MMSE scores, visuospatial performance and naming ability.

A detailed analysis of structural changes in thalamic subnuclei in PD has not been previously reported, but right-sided pulvinar dysfunction has been implicated in depressive illness, more generally, outside of the context of PD^14,15^. Interestingly, treatment of major depressive disorder with duloxetine, a potentially effective antidepressant in PD depression^26^, strengthens functional connectivity between the right pulvinar and right orbitofrontal cortex and also between the right pulvinar and the limbic regions of the right anterior cingulate cortex, left dorsomedial prefrontal cortex and left temporal gyrus^15^. These regions, along with the default mode network, to which the right pulvinar is connected regulate affective control and are implicated in mood disorders^27–29^. Intriguingly, we found that antidepressant use was associated with higher right PuA and PuM volumes at follow-up. Given that PD depression is likely to arise from disruption and degeneration of neural networks implicated in non-PD depression^5^, our findings, in conjunction with the broader literature, implicate the right PuA as a subnucleus of interest in the aetiology of PD depression.

Previous work has implicated the pulvinar in emotional processing^30^. As expected we found an association between anxiety and depression scores. However, anxiety scores were not associated with lower right pulvinar volumes, including the PuA and PuM, suggesting that the structural changes attributed to depression were not a pseudo-correlation secondary to anxiety. Similarly, we demonstrated that our findings of lower PuA volume being associated with depression scores are not confounded by levels of global cognition. However, the relationship between depression, cognitive impairment and structural change in Parkinson’s disease remains an important question that requires further specifically designed studies.

The severity of PD depression was associated with reduced FC across the majority of thalamic nuclei suggesting widespread white matter tract atrophy amongst fibres connected to the thalamus. Previous work has highlighted depression as a marker of PD disease severity^31^. Therefore, the association between depression scores and widespread white matter atrophy of tracts involving thalamic nuclei may reflect greater disease severity. Indeed, alpha-synuclein deposition and neuronal loss have been consistently demonstrated in thalamic nuclei in PD and are likely to be correlated with disease severity^32,33^.

In contrast, we saw increased FC after follow-up associated with higher depression scores in the bilateral VM and Pc nuclei. The VM nuclei have uniquely strong and diffuse interconnections to widespread cortical regions including the prefrontal cortex, enabling them to dynamically synchronise cortical networks^34^. It is plausible that white matter hypertrophy in the VM nuclei represents a compensatory mechanism to offset dysregulated neural circuits in brain regions implicated in PD depression, for example, the pulvinar-cortical networks. Similarly, white matter hypertrophy of tracts originating from the Pc may represent neural plasticity in response to aberrant neural circuity in affective control pathways involving the prefrontal cortex. However, the posterior probability distributions associated with the Pc and VM subnuclei segmentations were among the lowest across all subnuclei bilaterally (Supplementary Fig. 1). As such, it is possible that the volumetric and tract estimations derived from these segmentations are less robust, making any conclusions linked to higher FC in these subnuclei tentative. Further work will be needed to validate these findings in PD depression in other cohorts.

Depression scores correlated significantly with poorer clinical features of PD including motor symptoms even after correcting for dopamine dose and disease duration. Similarly, depression scores were also associated with poorer MMSE, naming ability and visuospatial performance, after correcting for age. The relationship between depressive symptomatology and severity of motor symptoms is in keeping with several previous studies^31,35,36^. The exact mechanism by which depression influences motor functioning remains unclear although a recent study of de novo PD patients found that depressed patients already had more severe motor symptoms at baseline, suggesting that depression is a marker of disease severity. Interestingly, in that study, both depressed and non-depressed PD patients had similar striatal dopaminergic levels suggesting that in PD depression, factors other than dopamine levels could affect motor symptoms^37^. We found that depression was also associated with visuoperceptual deficits which have also been shown to be associated with widespread macrostructural white matter degeneration^23^. It is most likely that PD depression is linked with these other non-motor PD features due to a shared aetiology of accelerated and more widespread Lewy body deposition in brain regions beyond the midbrain dopaminergic neurons^38,39^.

There are some potential limitations to consider. Depression was measured using a single measure, the HADS. Using a combination of multiple tools to measure depression severity could strengthen validity and reliability. We examined relationships with depression severity scores, rather than separating depressed from non-depressed patients, due to the limited availability of clinical data on depression. Our cohort did not include patients with more severe depressive symptoms. Future work should examine thalamic changes in PD across the spectrum of depression severity, to determine whether these findings apply across this range, or mainly relate to milder depressive symptoms.

Recent work has highlighted sex as an important biological variable that influences the phenotypic expression of PD, including mood symptoms, and responses to treatment^40^. In our study, we tried to account for sex differences. For example, there were no significant gender differences in either the PD or healthy control group and gender was corrected for in statistical analysis evaluating the association between depression and both thalamic subnuclei volumes and mean fibre cross-sections of white matter tracts connected to each subnucleus. However, future studies need to be designed and adequately powered to evaluate whether potential differences in the neural correlates of PD depression and possible neural plasticity in response to antidepressant treatment are driven by biological sex.

In our study, the detailed characterisation of antidepressant use was limited. It is possible that some effects also relate to premorbid use of antidepressants or other factors, such as dose or duration of treatment or class of antidepressant. Future work should specifically explore whether any potential protective effects of antidepressants relate to these factors.

Participants with other pathologies such as hypertension that could influence white matter structure and potentially affect FC values were not excluded; and similar to previous studies using fixel-based analysis (FBA), white matter hyperintensities were not specifically quantified or controlled for^41^. Finally, participants underwent neuroimaging whilst taking their regular dopaminergic medication, although it would be unlikely for this to affect structural integrity measures^42^.

In conclusion, our study utilised a novel thalamic segmentation tool to investigate volume and white matter macrostructural changes associated with depressive symptomatology in PD. Novel findings include PD depression being associated with right PuA volume loss and widespread thalamic white matter macrostructural loss as well as antidepressant use being associated with higher right PuA volume. Future work should examine PD patients with more severe depression and over a longer follow-up. In the longer term, a better understanding of the neural correlates of PD depression may reveal potential therapeutic targets.

## Methods

### Participants

The study included 76 people with PD, who had been recruited to our UK centre from affiliated clinics, and 26 age-matched controls, who were spouses or recruited from a volunteer database. PD participants were recruited to the study consecutively and only excluded if they had a history of traumatic brain injury; major co-morbid psychiatric or neurological disorder; contraindication to MRI; or PD duration of more than 10 years. PD participants satisfied the Queen Square Brain Bank PD diagnostic criteria. This cohort has been described in previous work from our group examining changes relating to cognition and hallucinations^23,24^. Ethical approval was received from the Queen Square Ethics Committee (reference no. 15.LO.0476) and all participants provided written informed consent.

### Clinical assessment

Clinical assessment was undertaken to evaluate symptoms relating to depression and anxiety as well as cognitive and disease-specific measures. Depression and anxiety severity was measured using the HADS^43^, which has previously been validated for use in patients with PD^44^. Cognitive testing included MMSE and MoCA as measures of global cognition, as well as the Stroop, digit span, verbal fluency, GNT, Hooper Visual Organisation Test (HVOT), logical memory recall and JLO. Disease-specific measures included the Movement Disorders MDS-UPDRS which measures motor and non-motor aspects of disease severity, UM-PDHQ evaluated hallucinations and RBDSQ assessed sleep. Levodopa dose equivalence scores (LEDD) were calculated as described previously^45^.

Clinical assessments, including neuroimaging (see below), were undertaken at baseline and followed up between 14 and 20 months later (mean = 15.4 months).

### MRI data acquisition

All participants were scanned on a 3 T Siemens Magnetom Prisma scanner (Siemens, Munich, Germany) with a 64-channel head coil. Diffusion-weighted imaging (DWI) was acquired with the following parameters: *b* = 50 s/mm^2^/17 directions, *b* = 300 s/mm^2^/8 directions, *b* = 1000 s/mm^2^/64 directions, *b* = 2000 s/mm^2^/64 directions, 2 × 2 × 2 mm isotropic voxels, echo time: 3260 ms, repetition time: 58 ms, 72 slices, 2 mm thickness and acceleration time factor of 2. The acquisition time for DWI was approximately 10 min. *T*1-weighted data were acquired using whole head 3D magnetisation prepared rapid acquisition gradient echo (MPRAGE) with the following parameters: voxel size 1 mm^3^, echo time: 3.34 ms, repetition time: 2530 ms, flip angle: 7°.

### Thalamic segmentation

Image processing used the “recon-all” function in FreeSurfer v6.0.0 (http://www.freesurfer.net). This includes motion correction, normalisation of signal intensity, skull stripping, Talaraich correction, and automated segmentation of subcortical white matter and grey matter (GM) structures^46^.

**Fig. 2:**
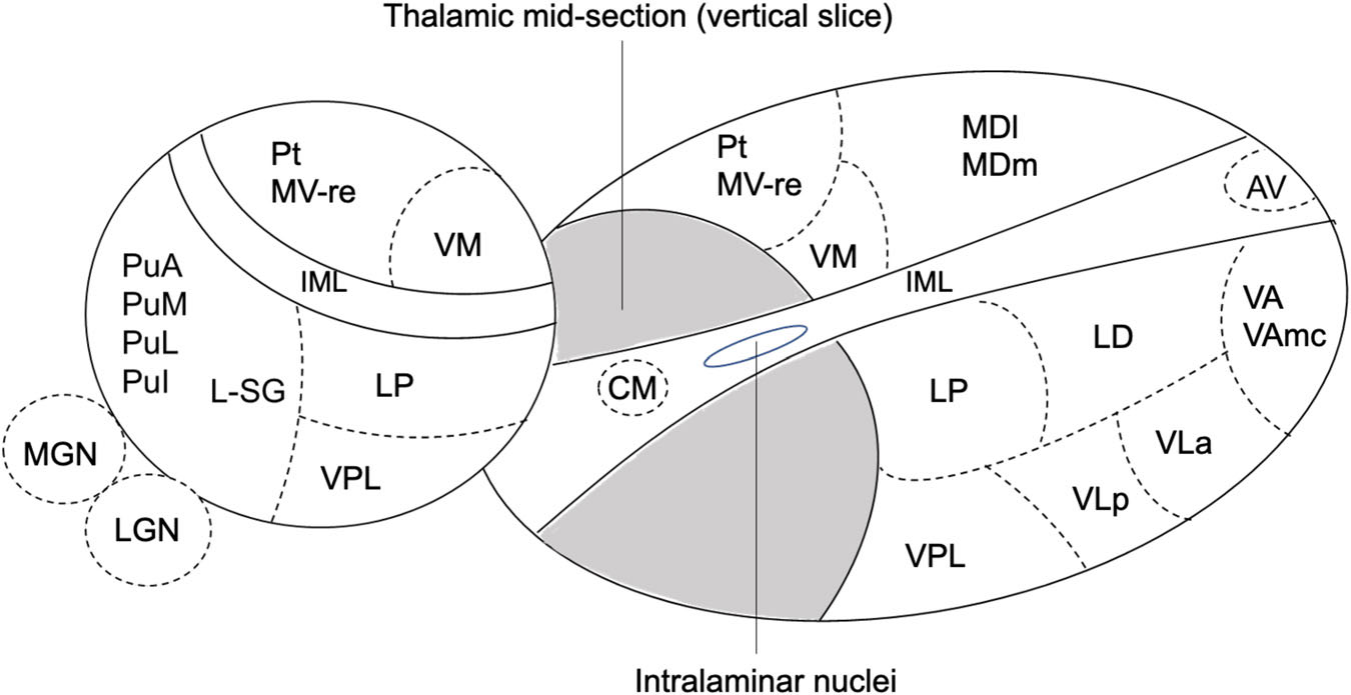
Three-dimensional schematic representation of thalamic subnuclei. The thalamus is divided into two by a vertical slice to reveal the medial surface. Abbreviations: Anterior nuclei (AV anteroventral); Lateral nuclei (LD laterodorsal, LP lateral posterior); Ventral nuclei (VA ventral anterior,VAmc ventral anterior magnocellular, VLa ventral lateral anterior, VLp ventral lateral posterior, VPL ventral posterolateral, VM ventromedial; Intralaminar nuclei (CM centromedian, CeM central medial, CL central lateral, Pc paracentral, Pf parafascicular);Medial nuclei (Pt paratenial, MV-re reuniens (medial ventral), MDm mediodorsal medial magnocellular, MDl mediodorsal lateral parvocellular); Posterior nuclei (LGN lateral geniculate nucleus, MGN medical geniculate nucleus, L-SG limitans (suprageniculate), PuA pulvinar anterior, PuM pulvinar medial, PuL pulvinar lateral, PuI pulvinar inferior); IML Internal medullary lamina.

The processed image files were further processed with the thalamic segmentation technique^22^ recently released with FreeSurfer (version 7.1.1). In brief, this method is a Bayesian segmentation algorithm based on a histologically derived probabilistic thalamic atlas, which divides each thalamus into 25 subnuclei (Fig. 2). We also extracted the posterior probability distributions associated with each thalamic nucleus segmentation, i.e., probabilities that every voxel in the image belongs to each of the nuclei according to the model (also known as “soft segmentations”).

### DWI preprocessing

DWIs were denoised and corrected for Gibbs ringing, eddy currents, motion, and bias field using MRtrix3 (mrtrix.org)^47–49^. Motion and distortion correction was performed using the dwipreproc pipeline in MRtrix which performs the following corrections: (1) EPI distortion correction^50^ using two b0 images, one acquired in the phase-encoded direction (PE) and one in the reversed direction; (2) B0-field inhomogeneity correction using FSL’s topup tool^51^; (3) Eddy-current and movement distortion correction^52^ using FSL’s eddy tool. This shows better performance than previous methods^53^. In addition, DWI spatial resolution of DWIs was upsampled using cubic interpolation to a voxel size of 1.3 mm^3^ to improve anatomical contrast and downstream template building, registration and statistics^54^. Intensity normalisation was then performed across participants to increase anatomic delineation and improve statistics^55^. We then computed fibre-orientation distributions (FODs) for each participant using multi-shell three-tissue constrained spherical-deconvolution, using the average response function for each tissue type (grey, white-matter, CSF)^56^. A group-averaged template was created from baseline data in 30 randomly selected subjects (20 PD, 10 controls), and each participant’s FODs were registered to this template^57^.

### Fibre cross-section

Classical diffusion tensor imaging techniques cannot model crossing fibres that are present in up to 90% of white matter voxels^57–59^. FBA is an emerging framework that uses a higher-order diffusion model that estimates the orientations of each fibre population and also quantifies degenerative changes in specific fibre populations within voxels. This allows comparisons of specific tracts (or ‘fixels’, specific fibre populations within voxels), instead of comparing measures that are averaged across voxels. It provides information about fibre morphology as well as fibre density. Here, we focused on fibre cross-section (FC) which is a relative metric of differences in fibre bundle cross-section compared to a template based on the study population and is thought to be a measure of white matter macrostructure^60^. We used this metric as our previous work in PD has shown FC to be a more sensitive measure of degeneration in PD, compared to other fixel-based measures such as fibre density and combined fibre density and cross-section^23^. FC was estimated for each fixel by calculating the distortion in the fibre bundle cross-section required to warp the subject image to the template image.

To assess white matter tracts connected to thalamic subnuclei, we generated tracts connected to each of the 50 thalamic subnuclei as follows: we used NiftytReg^61^ to register each subnucleus to the population template using affine linear registration. Next, we generated a tractogram for each thalamic subnucleus using probabilistic tractography on the population^54^. We initiated streamlines within each thalamic subnucleus to the ipsilateral hemisphere, whilst excluding the rest of the thalamus (to avoid overlap between tracts). This allowed us to generate a single tract-of-interest for each thalamic subnucleus to the cortex and mean FC could then be calculated for each tract of interest in every participant.

### Statistical analysis

Differences in demographics, cognitive test scores and disease-related measures were compared between participants with PD and HC. Independent sample *t*-tests and Mann–Whitney *U* tests were used for normally and non-normally distributed variables respectively, and *χ*^2^ for categorical variables. The Schapiro–Wilk test was used to assess normality. Statistical significance was set at *p* < 0.05. Statistical analyses were performed in R Version 4.0.3 and Python 3 with Jupyter Notebook version 5.5.0.

We evaluated the association of depression with other clinical features of PD because particular thalamic subnuclei have been implicated in the neurobiology of certain PD symptoms, for example, the mediodorsal and intralaminar nuclei in cognition and ventral nuclei in motor symptoms^62,63^. Therefore, if depression was found to be associated both with volume or white matter macrostructure changes of particular subnuclei as well as a clinical feature, which in turn is associated with changes in those same subnuclei, this could raise the important possibility of a pseudo-correlation.

To analyse the association of depression, which was measured by the average HADS depression score at baseline and follow up, with clinical features of PD we used a general linear mixed model (GLMM). The clinical measure of interest was the dependent variable and average HADS depression score, the independent variable, with time as a co-variate and participant as a random effect. For cognitive measures, age was an additional covariate while for disease-specific measures, LEDD and disease duration were additional covariates. A FDR correction was performed for 19 clinical measures, using the Benjamini–Hochberg method^64^.

Thalamic nuclei volumes and the mean FC of fibre bundles connected to thalamic subnuclei were computed. Two separate regression models were used to evaluate the relationship between HADS-depression scores and thalamic subnuclei volume and FC for each subnucleus in PD participants. In these models, subnuclei volume or mean FC were the dependent variables. For a cross-sectional evaluation at baseline, we used GLMM with baseline HADS depression score as the independent variable, age, intracranial volume and gender as co-variates, and participant as a random effect. In a model to evaluate whether HADS depression scores were associated with follow-up nuclei volumes and FC, we used a GLMM with an average HADS depression score derived from baseline and follow-up visit scores, as the independent variable. Subnuclei volumes and mean FC values were the dependent variable in these models which also had time between visits, age, intracranial volume and gender as co-variates, and participant as a random effect. A FDR correction was performed for 50 thalamic subnuclei tested for both volume and FC analyses, using the Benjamini–Hochberg method^64^.

We evaluated the effect of antidepressant use on measures of volume and FC in the mediodorsal and pulvinar nuclei bilaterally by adding antidepressant use as a covariate to the GLMM outlined above.

Post-hoc, we performed an additional GLMM analysis to evaluate whether our finding of depression being significantly associated with lower right PuA volume was influenced by depression also being associated with poorer cognitive performance. In this additional GLMM, we additionally corrected for MoCA scores, as well as age, intracranial volume and gender. We tested the bilateral pulvinar, MDm and MDl subnuclei using this GLMM because these nuclei have previously been implicated in PD depression or modulation by antidepressant therapy.

We further evaluated the posterior probability distributions (soft segmentations) associated with the subnuclei. For each nucleus, the probability distributions were thresholded at *p* > 0.1 and mean posterior probabilities for each subject were extracted. These were adjusted for age, total intracranial volume and gender using multiple linear regression, then averaged across subjects. This metric is of interest as the posterior probability distributions give an indication of the reliability of the individual subnuclei segmentations, and hence the relative reliability of any results derived from them.

### Missing data

Not all PD participants attempted digit span and logical memory tasks (*n* = 59) as these cognitive tests were added partway through data collection to optimise cognitive assessment. For particular cognitive tests, participants were excluded if they could not tolerate testing and this is highlighted in the “Results” section. Analyses were performed on available data without imputation.

### Reporting summary

Further information on research design is available in the Nature Research Reporting Summary linked to this article.

## Supplementary information

Supplementary material_Thalamic white matter macrostructure and subnuclei volumes in Parkinson’s disease depression

Reporting Summary

## Data Availability

Imaging and clinical data used in this study will be shared upon reasonable request to the corresponding author. All data and statistics generated from this study are presented in the manuscript and supplementary data.

## Code availability

All code used in this study will be shared upon reasonable request to the corresponding author.
