# Supplementary material for: Thalamic white matter macrostructure and subnuclei volumes in Parkinson’s disease depression
Source: NPJ Parkinsons Dis. 2022 Jan 10;8:2. doi: 10.1038/s41531-021-00270-y (PMC8748828; doi:10.1038/s41531-021-00270-y)
Supplement: Supplementary file 1 — Supplementary material_Thalamic white matter macrostructure and subnuclei volumes in Parkinson’s disease depression [file 41531_2021_270_MOESM1_ESM.pdf]

Supplementary Material

Title

Thalamic white matter macrostructure and subnuclei volumes in Parkinson's disease depression

Authors

Bhome R[1], Zarkali A[1], Thomas GEC[1], Iglesias JE[2,3,4], Cole JH[1,2], Weil RS[1,5,6]

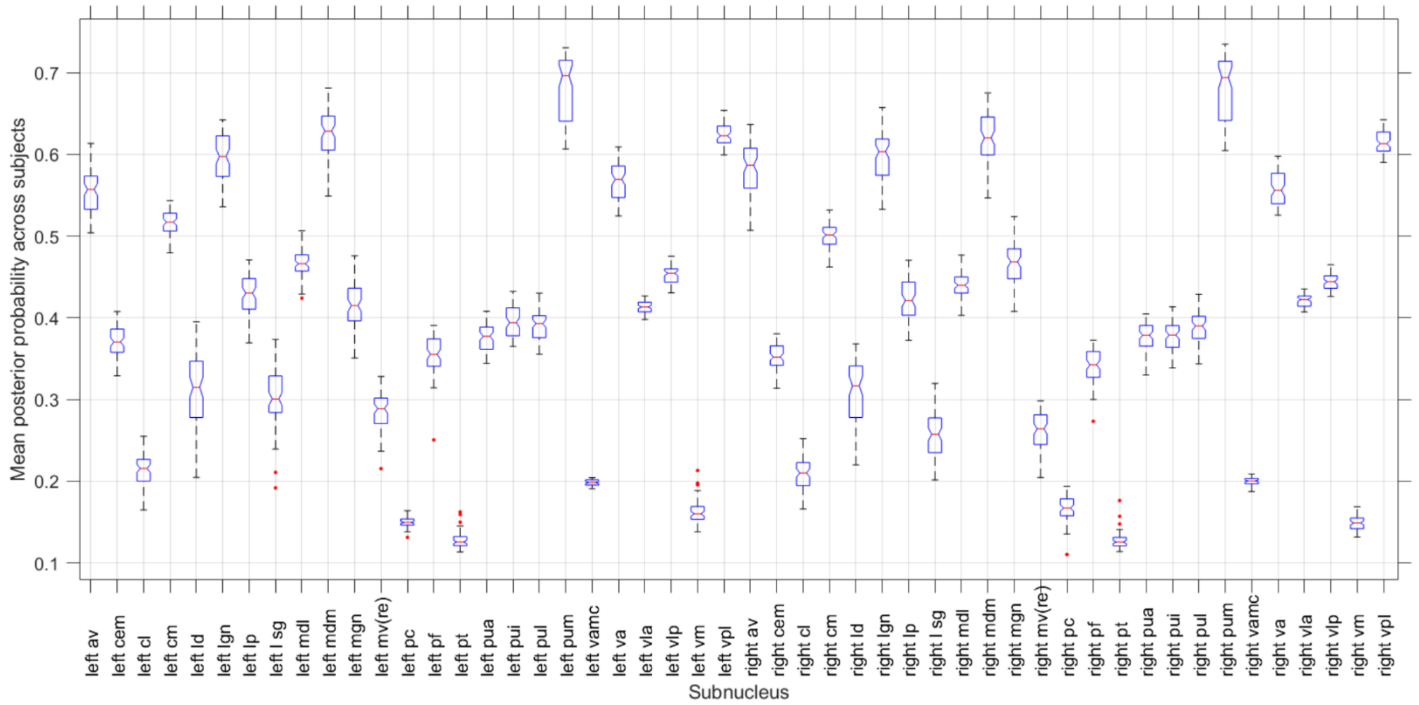

Supplementary figure 1. Subject mean posterior probabilities for voxels higher than p=0.1 in thalamic subnuclei soft segmentations. Median, IQR and range across subject means for each subnucleus segmentation are displayed. Outliers are displayed as red dots.

Anterior nuclei: (AV= anteroventral);

Lateral nuclei: (LD= laterodorsal, LP= = lateral posterior);

Ventral nuclei: (VA= ventral anterior, Vamc,= ventral anterior magnocellular , VLa= ventral lateral anterior, VLp= ventral lateral posterior, VPL= ventral posterolateral, VM= ventromedial;

Intralaminar nuclei: (CeM= central medial, CL= central lateral, Pc=paracentral, CM= centromedian Pf= parafascicular);

Medial nuclei: (Pt= paratenial, MV-re= reuniens (medial ventral), MDm= mediodorsal medial magnocellular, MDl= mediodorsal lateral parvocellular)

Posterior nuclei: (LGN=lateral geniculate, MGN= medical geniculate, L-SG= limitans (suprageniculate), PuA= pulvinar anterior, PuM= pulvinar medial, PuL= pulvinar lateral, PuI= pulvinar inferior).

Supplementary Table 1. Association between baseline HADS depression score and thalamic subnuclei volume								
	**Left Thalamus**				**Right Thalamus**			
**Nuclei**	**Baseline Volume (mm^3)**	**Adjusted beta (SE)**	**95% CI**	**p value[a]**	**Baseline Volume (mm^3)**	**Adjusted beta (SE)**	**95% CI**	**p value[a]**
**Anterior group**								
AV	126.76 (15.77)	0.11 (0.49)	-0.86 – 1.07	0.83	141.07 (18.03)	-0.17 (0.61)	-1.36 – 1.03	0.79
**Lateral group**								
LD	27.33 (7.29)	0.05 (0.27)	-0.49 – 0.58	0.87	26.20 (5.76)	0.30 (0.21)	-0.11 – 0.71	0.16
LP	122.09 (15.51)	-0.27 (0.48)	-1.21 – 0.66	0.57	109.67 (13.66)	0.58 (0.42)	-0.25 – 1.41	0.17
**Ventral group**								
VA	381.10 (46.16)	-0.43 (1.13)	-2.66 – 1.79	0.70	392.75 (46.23)	-2.07 (1.21)	-4.43 – 0.29	0.09
VAmc	27.91 (3.56)	-0.09 (0.10)	-0.28 – 0.10	0.35	29.35 (3.68)	-0.12 (0.10)	-0.32 – 0.07	0.22
VLa	594.93 (69.07)	-1.71 (1.69)	-5.02 – 1.60	0.31	616.26 (69.72)	-2.99 (1.80)	-6.52 – 0.54	0.10
VLp	772.62 (84.59)	-2.91 (2.25)	-7.33 – 1.50	0.20	793.23 (86.38)	-3.07 (2.27)	-7.52 – 1.37	0.18
VPL	800.84 (95.13)	-3.44 (2.65)	-8.63 – 1.75	0.19	807.80 (99.60)	-1.77 (2.74)	-7.13 – 3.59	0.52
VM	18.94 (2.35)	-0.05 (0.07)	-0.18 – 0.09	0.51	19.62 (2.65)	-0.04 (0.08)	-0.18 – 0.11	0.64
**Intralaminar group**								
CeM	58.50 (8.40)	-0.16 (0.28)	-0.71 – 0.40	0.58	62.14 (8.92)	-0.33 (0.30)	-0.92 – 0.26	0.27
CL	36.53 (6.40)	0.28 (0.23)	-0.17 – 0.73	0.22	37.08 (5.02)	0.19 (0.18)	-0.16 – 0.54	0.29
Pc	3.06 (0.48)	-0.01 (0.01)	-0.04 – 0.02	0.42	3.15 (0.51)	-0.03 (0.02)	-0.06 – 0.004	0.10
CM	241. 14 (28.54)	-0.04 (0.02)	-0.05 – 0.04	0.87	241.29 (27.40)	-0.53 (0.76)	-2.00 – 0.96	0.49
Pf	53.49 (6.58)	0.15 (0.19)	-0.23 – 0.52	0.44	55.24 (6.05)	0.15 (0.17)	-0.19 – 0.49	0.39
**Medial group**								
Pt	6.80 (0.79)	-0.004 (0.02)	-0.05 – 0.04	0.87	6.63 (0.69)	-0.001 (0.02)	-0.04 – 0.04	0.94
MV-re	9.63 (1.85)	-0.02 (0.06)	-0.15 – 0.10	0.74	9.81 (2.04)	-0.06 (0.07)	-0.20 – 0.08	0.40
MDm	624.67 (82.33)	-4.63 (2.42)	-9.38 – 0.12	0.06	649.59 (82.40)	-3.21 (2.42)	-7.95 – 1.54	0.19
MDl	245.00 (27.60)	-1.46 (0.80)	-3.02 – 0.11	0.07	260.49 (25.22)	-0.82 (0.71)	-2.20 – 0.56	0.25
**Posterior group**								
LGN	158.63 (27.79)	-1.32 (0.80)	-2.88 – 0.24	0.10	170.08 (27.59)	-0.20 (0.80)	-1.77 – 1.37	0.80
MGN	114.02 (18.71)	-0.69 (0.60)	-1.87 – 0.49	0.25	114.23 (18.05)	-0.82 (0.52)	-1.83 – 0.20	0.12
L-SG	24.69 (5.15)	-0.05 (0.17)	-0.38 – 0.28	0.77	23.78 (5.18)	0.05 (0.16)	-0.27 – 0.36	0.78
PuA	194.63 (21.69)	-0.29 (0.57)	-1.41 – 0.83	0.61	214.48 (23.29)	-0.77 (0.62)	-1.99 – 0.45	0.21

PuM	939.30 (105.19)	-3.05 (3.10)	-9.13 – 3.03	0.33	979.63 (106.99)	-3.28 (3.19)	-9.53 – 2.97	0.30
PuL	183.23 (30.12)	-0.16 (0.95)	-2.02 – 1.71	0.87	205.28 (31.97)	-0.79 (1.00)	-2.75 – 1.17	0.43
PuI	171.55 (23.62)	-0.61 (0.69)	-1.97 – 0.76	0.38	189.12 (23.49)	-0.27 (0.73)	-1.69 – 1.16	0.72

Anterior nuclei: (AV= anteroventral);
Lateral nuclei: (LD= laterodorsal, LP= = lateral posterior);
Ventral nuclei: (VA= ventral anterior, Vamc,= ventral anterior magnocellular , VLa= ventral lateral anterior, VLp= ventral lateral posterior, VPL= ventral posterolateral, VM= ventromedial;
Intralaminar nuclei: (CeM= central medial, CL= central lateral, Pc=paracentral, CM= centromedian Pf= parafascicular);
Medial nuclei: (Pt= paratenial, MV-re= reuniens (medial ventral), MDm= mediodorsal medial magnocellular, MDl= mediodorsal lateral parvocellular)
Posterior nuclei: (LGN=lateral geniculate, MGN= medical geniculate, L-SG= limitans (suprageniculate), PuA= pulvinar anterior, PuM= pulvinar medial, PuL= pulvinar lateral, PuI= pulvinar inferior).
[a]P values were analysed by a general linear model corrected by age, gender, total intracranial volume at baseline
There were no significant associations at baseline

Supplementary Table 2. Association of HADS depression score with thalamic subnuclei volume with global cognition included as an additional covariate[a]

	Left Thalamus				Right Thalamus			
**Nuclei group**	**Baseline volume (mm^3)[b]**	**Longitudinal change (mm3)[b]**	**beta**	**p value[c]**	**Baseline volume (mm^3)[b]**	**Longitudinal change (mm^3)[b]**	**beta**	**p value[c]**
MDm	624.67 (82.33)	-9.78 (43.98)	-3.54 (2.40)	0.14	649.59 (82.40)	-12.64 (56.65)	-3.75 (2.72)	0.17
MDl	245.00 (27.60)	-0.93 (15.29)	-0.80 (0.70)	0.30	260.49 (25.22)	-0.81 (14.00)	-1.05 (0.73)	0.15
PuA	194.63 (21.69)	-4.16 (10.70)	-0.31 (0.58)	0.60	214.48 (23.29)	-2.02 (14.00)	-1.58 (0.59)	**0.007**
PuM	939.30 (105.19)	-12.68 (48.58)	-3.00 (3.28)	0.36	979.63 (106.99)	-3.65 (57.17)	-7.18 (3.12)	0.02
PuL	183.23 (30.12)	-0.88 (15.15)	-0.22 (1.01)	0.83	205.28 (31.97)	3.64 (15.30)	-0.99 (1.05)	0.34
PuI	171.55 (23.62)	-2.11 (16.32)	-0.25 (0.75)	0.73	189.12 (23.49)	-1.05 (18.81)	-0.63 (0.75)	0.40

MDm= mediodorsal medial magnocellular, MDl= mediodorsal lateral parvocellular
PuA= pulvinar anterior, PuM= pulvinar medial, PuL= pulvinar lateral, PuI= pulvinar inferior.
[a]n=75
[b]For each nucleus, baseline volume and longitudinal change are presented as mean (SD).
[c]p values were analysed by a GLMM corrected by age, gender total intracranial volume, time between visits and average MOCA scores (derived from baseline and follow up scores) as co-variates, with participant as a random effect.

Supplementary Table 3. Association of HADS anxiety score with thalamic subnuclei volume								
	Left Thalamus				Right Thalamus			
**Nuclei group**	**Baseline volume (mm^3)[a]**	**Longitudinal change (mm3)[a]**	**beta**	**p value[b]**	**Baseline volume (mm^3)[a]**	**Longitudinal change (mm^3)[a]**	**beta**	**p value[b]**
**Anterior**								
AV	126.76 (15.77)	-0.69 (7.74)	0.10	0.82	141.07 (18.03)	0.21 (8.29)	-0.60	0.25
**Lateral**								
LD	27.33 (7.29)	-0.01 (2.69)	0.14	0.57	26.20 (5.76)	0.17 (3.30)	0.12	0.55
LP	122.09 (15.51)	-0.24 (8.89)	0.18	0.66	109.67 (13.66)	0.54 (8.23)	0.55	0.14
**Ventral**								
VA	381.10 (46.16)	-2.02 (23.49)	-0.34	0.72	392.75 (46.23)	-1.50 (21.64)	-1.41	0.18
VAmc	27.91 (3.56)	-0.35 (2.18)	-0.02	0.82	29.35 (3.68)	-0.11 (2.00)	-0.12	0.14
VLa	594.93 (69.07)	-5.39 (32.26)	-1.57	0.30	616.26 (69.72)	-0.94 (29.77)	-1.25	0.42
VLp	772.62 (84.59)	-7.12 (40.77)	-2.11	0.72	793.23 (86.38)	-2.52 (37.12)	-0.76	0.70
VPL	800.84 (95.13)	-13.97 (50.60)	-0.08	0.97	807.80 (99.60)	-5.27 (41.80)	0.05	0.98
VM	18.94 (2.35)	-0.30 (1.27)	0.03	0.54	19.62 (2.65)	-0.17 (1.21)	-0.02	0.74
**Intralaminar**								
CeM	58.50 (8.40)	-0.45 (5.35)	-0.05	0.85	62.14 (8.92)	0.09 (4.71)	-0.16	0.57
CL	36.53 (6.40)	0.13 (3.71)	0.16	0.44	37.08 (5.02)	0.18 (3.89)	0.12	0.46
Pc	3.06 (0.48)	-0.05 (0.29)	-0.006	0.65	3.15 (0.51)	-0.09 (0.28)	-0.02	0.22
CM	241. 14 (28.54)	-2.26 (14.77)	0.36	0.60	241.29 (27.40)	-1.79 (14.05)	-0.36	0.57
Pf	53.49 (6.58)	-0.32 (3.84)	0.24	0.15	55.24 (6.05)	0.01 (3.73)	0.009	0.96
**Medial**								
Pt	6.80 (0.79)	-0.003 (0.41)	0.01	0.60	6.63 (0.69)	0.01 (0.38)	0.01	0.46
MV-re	9.63 (1.85)	-0.05 (1.41)	-0.04	0.48	9.81 (2.04)	-0.02 (1.54)	-0.02	0.75
MDm	624.67 (82.33)	-9.78 (43.98)	-0.19	0.93	649.59 (82.40)	-12.64 (56.65)	-1.82	0.43
MDl	245.00 (27.60)	-0.93 (15.29)	-0.43	0.53	260.49 (25.22)	-0.81 (14.00)	-1.18	0.05
**Posterior**								
LGN	158.63 (27.79)	-2.81 (14.03)	0.009	0.99	170.08 (27.59)	0.43 (13.89)	0.56	0.41
MGN	114.02 (18.71)	-2.12 (11.30)	-0.06	0.91	114.23 (18.05)	-1.43 (9.89)	0.11	0.79
L-SG	24.69 (5.15)	0.64 (3.55)	-0.11	0.42	23.78 (5.18)	0.22 (3.50)	-0.01	0.91
PuA	194.63 (21.69)	-4.16 (10.70)	0.11	0.83	214.48 (23.29)	-2.02 (14.00)	-0.37	0.47
PuM	939.30 (105.19)	-12.68 (48.58)	1.39	0.62	979.63 (106.99)	-3.65 (57.17)	-1.81	0.51
PuL	183.23 (30.12)	-0.88 (15.15)	-0.25	0.77	205.28 (31.97)	3.64 (15.30)	-0.08	0.93
PuI	171.55 (23.62)	-2.11 (16.32)	0.42	0.51	189.12 (23.49)	-1.05 (18.81)	-0.01	0.99

Anterior nuclei: (AV= anteroventral);
Lateral nuclei: (LD= laterodorsal, LP= = lateral posterior);

Ventral nuclei: (VA= ventral anterior, Vamc,= ventral anterior magnocellular , VLa= ventral lateral anterior, VLp= ventral lateral posterior, VPL= ventral posterolateral, VM= ventromedial;
Intralaminar nuclei: (CeM= central medial, CL= central lateral, Pc=paracentral, CM= centromedian Pf= parafascicular);
Medial nuclei: (Pt= paratenial, MV-re= reuniens (medial ventral), MDm= mediodorsal medial magnocellular, MDl= mediodorsal lateral parvocellular)
Posterior nuclei: (LGN=lateral geniculate, MGN= medical geniculate, L-SG= limitans (suprageniculate), PuA= pulvinar anterior, PuM= pulvinar medial, PuL= pulvinar lateral, PuI= pulvinar inferior).
[a]For each nucleus, baseline volume and longitudinal change are presented as mean (SD).
[b]p values were analysed by a GLMM corrected by age, gender total intracranial volume, time between visits with participant as a random effect.

Supplementary Table 4. Characteristics of PD participants taking and not taking antidepressant medication

Attribute	Antidepressant (n=9)	No antidepressant (n=67)	Statistic
**Demographics**			
Age, y	60.56 (6.47)	65.12 (8.01)	t=-1.62, p=0.11
Male, n (%)	3 (33%)	67 (88)	χ2=1.11, p=0.29
**Mood (HADS)[a]**			
Depression score	5.39 (4.38)	3.92 (2.99)	U=359.0, p=0.36
Anxiety score	8.94 (5.90)	5.01 (2.96)	U= 469.5, p=0.007
**Cognitive testing**			
MMSE	29.00 (1.15)	29.01 (1.18)	U=299.0, p=0.97
MoCA	28.11 (1.91)	27.93 (2.23)	U=314.5, p=0.84
GNT	24.67 (2.94)	24.04 (2.52)	U=356.0, p=0.38
JLO[b]	24.22 (3.74)	24.90 (3.95)	t=-0.49, p=0.63
**Disease specific measures**			
Disease duration, y	3.56 (2.01)	4.19 (2.53)	U=260.5, p=0.51
LEDD	420.56 (178.43)	428.83 (224.61)	t=-0.10, p=0.92
MDS-UPDRS	46.89 (13.94)	45.73 (21.78)	U=329.5, p=0.66
MDS-UPDRS Motor Score	25.67 (10.55)	23.07 (12.52)	U=352.5, p=0.42
RBDSQ	5.33 (3.02)	4.00 (2.07)	U=370.5, p=0.27
UM-PDHQ	2.22 (3.29)	0.48 (1.44)	U=372.0, p=0.08

Abbreviations: HADS = Hospital Anxiety and Depression Scale; MMSE = Mini-Mental State Examination; MoCA = Montreal Cognitive Assessment; GNT = Graded Naming Test; = Judgment of Line Orientation; LEDD = Total levodopa equivalent dose; MDS-UPDRS = Movement Disorders Society Unified Parkinson's Disease Rating Scale; RBDSQ = REM Sleep Behaviour Disorder Screening Questionnaire; UM-PDHQ = University of Miami Hallucinations Questionnaire

All data shown are mean (SD) except gender.

[a]Average HADS depression and anxiety scores derived from baseline and follow up scores

[b] No antidepressant (n=66)

Supplementary Table 5. Association between baseline HADS depression score and thalamic subnuclei FC score								
	**Left Thalamus**				**Right Thalamus**			
**Nuclei**	**Baseline FC**	**Adjusted beta (SE)**	**95% CI**	**p value[a]**	**Baseline FC**	**Adjusted beta (SE)**	**95% CI**	**p value[a]**
**Anterior group**								
AV	0.071 (0.025)	-0.002 (0.001)	-0.004 – 0.001	0.21	0.078 (0.064)	-0.002 (0.001)	-0.004 – 0.001	0.18
**Lateral group**								
LD	0.085 (0.065)	-0.002 (0.001)	-0.004 – 0.001	0.16	0.083 (0.065)	-0.002 (0.001)	-0.004 – 0.001	0.16
LP	0.016 (0.054)	-0.000 (0.001)	-0.003 – 0.003	0.79	0.075 (0.063)	-0.002 (0.001)	-0.004 – 0.001	0.14
**Ventral group**								
VA	0.070 (0.065)	-0.001 (0.001)	-0.004 – 0.001	0.35	0.071 (0.066)	-0.001 (0.001)	-0.004 – 0.001	0.29
VAmc	0.067 (0.059)	-0.001 (0.001)	-0.003 – 0.001	0.47	0.072 (0.063)	-0.001 (0.001)	-0.004 – 0.001	0.34
VLa	0.067 (0.061)	-0.001 (0.001)	-0.003 – 0.002	0.56	0.066 (0.062)	-0.001 (0.001)	-0.003 – 0.002	0.51
VLp	0.066 (0.061)	-0.001 (0.001)	-0.003 – 0.002	0.56	0.067 (0.061)	-0.001 (0.001)	-0.003 – 0.002	0.63
VPL	0.069 (0.059)	-0.001 (0.001)	-0.003 – 0.002	0.60	0.065 (0.058)	-0.000 (0.001)	-0.002 – 0.002	0.83
VM	-0.300 (0.247)	0.002 (0.003)	-0.008 – 0.012	0.66	-0.287 (0.250)	0.003 (0.005)	-0.007 – 0.012	0.55
**Intralaminar group**								
CeM	0.065 (0.058)	-0.001 (0.001)	-0.003 – 0.002	0.56	0.068 (0.057)	-0.000 (0.001)	-0.002 – 0.002	0.92
CL	0.083 (0.065)	-0.002 (0.001)	-0.005 – 0.000	0.09	0.085 (0.074)	-0.003 (0.003)	-0.009 – 0.002	0.24
Pc	-0.217 (0.180)	0.004 (0.003)	-0.003 – 0.010	0.29	-0.227 (0.144)	0.004 (0.004)	-0003 – 0.011	0.27
CM	0.070 (0.060)	-0.001 (0.001)	-0.003 – 0.001	0.39	0.073 (0.060)	-0.001 (0.001)	-0.003 – 0.002	0.53
Pf	0.062 (0.057)	-0.001 (0.001)	-0.003 – 0.002	0.62	0.069 (0.057)	-0.000 (0.001)	-0.003 – 0.002	0.83
**Medial group**								
Pt	-0.217 (0.180)	0.004 (0.003)	-0.003 – 0.001	0.29	-0.227 (0.144)	0.004 (0.004)	-0.003 – 0.011	0.27
MV-re	0.069 (0.059)	-0.001 (0.001)	-0.003 – 0.001	0.42	0.074 (0.062)	-0.001 (0.001)	-0.004 – 0.001	0.30
MDm	0.039 (0.065)	-0.002 (0.001)	-0005 – 0.001	0.12	0.039 (0.057)	0.000 (0.002)	-0.003 – 0.003	0.92
MDl	0.016 (0.059)	-0.001 (0.001)	-0.004 – 0.002	0.50	-0.007 (0.073)	-0.001 (0.001)	-0.005 – 0.004	0.78
**Posterior group**								
LGN	0.081 (0.066)	-0.001 (0.001)	-0.004 – 0.001	0.25	0.074 (0.065)	-0.002 (0.001)	-0.004 – 0.001	0.16
MGN	0.080 (0.063)	-0.001 (0.001)	-0.004 – 0.001	0.28	0.077 (0.063)	-0.001 (0.001)	-0.004 – 0.001	0.24
L-SG	0.069 (0.065)	-0.002 (0.001)	-0.005 – 0.000	0.09	0.090 (0.066)	-0.002 (0.001)	-0.005 – 0.000	0.07
PuA	-0.016 (0.202)	-0.004 (0.005)	-0.014 – 0.007	0.50	0.047 (0.071)	-0.002 (0.002)	-0.006 – 0.001	0.15
PuM	0.084 (0.066)	-0.002 (0.001)	-0.004 – 0.001	0.16	0.080 (0.066)	-0.002 (0.001)	-0.004 – 0.001	0.14

PuL	0.076 (0.064)	-0.001 (0.001)	-0.003 – 0.001	0.39	0.072 (0.064)	-0.001 (0.001)	-0.004 – 0.001	0.35
PuI	0.083 (0.064)	-0.001 (0.001)	-0.004 – 0.001	0.25	0.081 (0.064)	-0.001 (0.001)	-0.004 – 0.001	0.23

Anterior nuclei: (AV= anteroventral);
Lateral nuclei: (LD= laterodorsal, LP= = lateral posterior);
Ventral nuclei: (VA= ventral anterior, Vamc,= ventral anterior magnocellular , VLa= ventral lateral anterior, VLp= ventral lateral posterior, VPL= ventral posterolateral, VM= ventromedial;
Intralaminar nuclei: (CeM= central medial, CL= central lateral, Pc=paracentral, CM= centromedian Pf= parafascicular);
Medial nuclei: (Pt= paratenial, MV-re= reuniens (medial ventral), MDm= mediodorsal medial magnocellular, MDl= mediodorsal lateral parvocellular)
Posterior nuclei: (LGN=lateral geniculate, MGN= medical geniculate, L-SG= limitans (suprageniculate), PuA= pulvinar anterior, PuM= pulvinar medial, PuL= pulvinar lateral, PuI= pulvinar inferior).
[a]P values were analysed by a general linear model corrected by age, gender, total intracranial volume at baseline
There were no significant associations at baseline
