# Supplementary material for: Thalamic white matter macrostructure and subnuclei volumes in Parkinson’s disease depression
Source: NPJ Parkinsons Dis. 2022 Jan 10;8:2. doi: 10.1038/s41531-021-00270-y (PMC8748828; doi:10.1038/s41531-021-00270-y)
Supplement: Supplementary file 2 — Reporting Summary [file 41531_2021_270_MOESM2_ESM.pdf]

nature portfolio

Corresponding author(s): Dr Rohan Bhome
Last updated by author(s): Nov 29, 2021

# Reporting Summary

Nature Portfolio wishes to improve the reproducibility of the work that we publish. This form provides structure for consistency and transparency in reporting. For further information on Nature Portfolio policies, see our Editorial Policies and the Editorial Policy Checklist.

## Statistics

For all statistical analyses, confirm that the following items are present in the figure legend, table legend, main text, or Methods section.

| n/a | Confirmed | |
|---|---|---|
| ☐ | ☒ | The exact sample size (*n*) for each experimental group/condition, given as a discrete number and unit of measurement |
| ☐ | ☒ | A statement on whether measurements were taken from distinct samples or whether the same sample was measured repeatedly |
| ☐ | ☒ | The statistical test(s) used AND whether they are one- or two-sided<br>*Only common tests should be described solely by name; describe more complex techniques in the Methods section.* |
| ☐ | ☒ | A description of all covariates tested |
| ☐ | ☒ | A description of any assumptions or corrections, such as tests of normality and adjustment for multiple comparisons |
| ☐ | ☒ | A full description of the statistical parameters including central tendency (e.g. means) or other basic estimates (e.g. regression coefficient) AND variation (e.g. standard deviation) or associated estimates of uncertainty (e.g. confidence intervals) |
| ☐ | ☒ | For null hypothesis testing, the test statistic (e.g. *F*, *t*, *r*) with confidence intervals, effect sizes, degrees of freedom and *P* value noted<br>*Give P values as exact values whenever suitable.* |
| ☒ | ☐ | For Bayesian analysis, information on the choice of priors and Markov chain Monte Carlo settings |
| ☐ | ☒ | For hierarchical and complex designs, identification of the appropriate level for tests and full reporting of outcomes |
| ☐ | ☒ | Estimates of effect sizes (e.g. Cohen's *d*, Pearson's *r*), indicating how they were calculated |

*Our web collection on statistics for biologists contains articles on many of the points above.*

## Software and code

Policy information about availability of computer code

| | |
|---|---|
| Data collection | No software was used. |
| Data analysis | No software was used. |

For manuscripts utilizing custom algorithms or software that are central to the research but not yet described in published literature, software must be made available to editors and reviewers. We strongly encourage code deposition in a community repository (e.g. GitHub). See the Nature Portfolio guidelines for submitting code & software for further information.

## Data

Policy information about availability of data

All manuscripts must include a data availability statement. This statement should provide the following information, where applicable:

- Accession codes, unique identifiers, or web links for publicly available datasets
- A description of any restrictions on data availability
- For clinical datasets or third party data, please ensure that the statement adheres to our policy

Imaging and clinical data used in this study will be shared upon reasonable request to the corresponding author. All data and statistics generated from this study are presented in the manuscript and supplementary data.

# Field-specific reporting

Please select the one below that is the best fit for your research. If you are not sure, read the appropriate sections before making your selection.

☒ Life sciences ☐ Behavioural & social sciences ☐ Ecological, evolutionary & environmental sciences

For a reference copy of the document with all sections, see nature.com/documents/nr-reporting-summary-flat.pdf

# Life sciences study design

All studies must disclose on these points even when the disclosure is negative.

| | |
|---|---|
| Sample size | The sample size was determined by the size of the existing dataset. Previous work had demonstrated that this sample size provided adequate power to robustly investigate longitudinal neuroimaging correlates of visual hallucinations and we expected that it would provide adequate power to investigate longitudinal neuroimaging correlates of depression, another neuropsychiatric feature of Parkinson's Disease. |
| Data exclusions | No data were excluded. Missing data arose due to some participants being unable to tolerate neuropsychological testing. |
| Replication | Going forward, it would be possible to replicate our work on a distinct Parkinson's disease cohort based on clear detailed methods included. |
| Randomization | In our study participants were not randomly allocated as we compared Parkinson's disease participants to healthy controls. Or compared associations of clinical characteristics within the Parkinson's disease cohort. |
| Blinding | Blinding was not relevant in our study as the study group of interest did not receive differing interventions. |

# Reporting for specific materials, systems and methods

We require information from authors about some types of materials, experimental systems and methods used in many studies. Here, indicate whether each material, system or method listed is relevant to your study. If you are not sure if a list item applies to your research, read the appropriate section before selecting a response.

## Materials & experimental systems

| n/a | Involved in the study | |
|---|---|---|
| ☒ | ☐ | Antibodies |
| ☒ | ☐ | Eukaryotic cell lines |
| ☒ | ☐ | Palaeontology and archaeology |
| ☒ | ☐ | Animals and other organisms |
| ☐ | ☒ | Human research participants |
| ☒ | ☐ | Clinical data |
| ☒ | ☐ | Dual use research of concern |

## Methods

| n/a | Involved in the study | |
|---|---|---|
| ☒ | ☐ | ChIP-seq |
| ☒ | ☐ | Flow cytometry |
| ☐ | ☒ | MRI-based neuroimaging |

## Human research participants

Policy information about studies involving human research participants

| | |
|---|---|
| Population characteristics | PD participants were recruited to the study consecutively and only excluded if they had a history of traumatic brain injury; major co-morbid psychiatric or neurological disorder; contraindication to MRI; or PD duration of more than ten years. PD participants satisfied the Queen Square Brain Bank PD diagnostic criteria. |
| Recruitment | Participants were recruited to our UK centre (National Hospital for Neurology and Neurosurgery) from affiliated clinics, and age-matched controls were spouses or recruited from a volunteer database. |
| Ethics oversight | Ethical approval was received from the Queen Square Ethics Committee (reference no. 15.LO.0476) |

Note that full information on the approval of the study protocol must also be provided in the manuscript.

## Magnetic resonance imaging

### Experimental design

| | |
|---|---|
| Design type | Structural (no task) |
| Design specifications | Structural |

Behavioral performance measures: Not task based

## Acquisition

Imaging type(s): Diffusion

Field strength: 3T

Sequence & imaging parameters: All participants were scanned on a 3T Siemens Magnetom Prisma scanner (Siemens, Munich, Germany) with a 64-channel head coil. Diffusion weighted imaging (DWI) was acquired with the following parameters: b = 50 s/mm2 / 17 directions, b = 300 s/mm2 / 8 directions, b = 1000 s/mm2 / 64 directions, b = 2000 s/mm2 / 64 directions, 2x2x2 mm isotropic voxels, echo time: 3260 ms, repetition time: 58 ms, 72 slices, 2 mm thickness and acceleration time factor of 2. Acquisition time for DWI was approximately 10 minutes. T1-weighted data were acquired using whole head 3D magnetization prepared rapid acquisition gradient echo (MPRAGE) with the following parameters: voxel size 1mm3, echo time: 3.34 ms, repetition time: 2530 ms, flip angle: 7°).

Area of acquisition: whole brain

Diffusion MRI: ☒ Used ☐ Not used

Parameters: Diffusion weighted imaging (DWI) was acquired with the following parameters: b = 50 s/mm2 / 17 directions, b = 300 s/mm2 / 8 directions, b = 1000 s/mm2 / 64 directions, b = 2000 s/mm2 / 64 directions, 2x2x2 mm isotropic voxels, echo time: 3260 ms, repetition time: 58 ms, 72 slices, 2 mm thickness and acceleration time factor of 2. Acquisition time for DWI was approximately 10 minutes.

## Preprocessing

Preprocessing software: DWIs were denoised and corrected for Gibbs ringing, eddy currents, motion, and bias field using MRtrix3 (mrtrix.org).Motion and distortion correction was performed using the dwipreproc pipeline in MRtrix which performs the following corrections: 1) EPI distortion correction using two b0 images one acquired in the phase encoded direction (PE) and one in the reversed direction, 2) B0-field inhomogeneity correction using FSL's topup tool, 3) Eddy-current and movement distortion correction using FSL's eddy tool. This shows better performance than previous methods. In addition, DWI spatial resolution of DWIs was upsampled using cubic interpolation to a voxel size of 1.3mm3 to improve anatomical contrast and downstream template building, registration and statistics. Intensity normalisation was then performed across participants to increase anatomic delineation and improve statistics.

Normalization: Intensity normalisation was then performed across participants to increase anatomic delineation and improve statistics.

Normalization template: Talaraich

Noise and artifact removal: 1) EPI distortion correction using two b0 images one acquired in the phase encoded direction (PE) and one in the reversed direction, 2) B0-field inhomogeneity correction using FSL's topup tool, 3) Eddy-current and movement distortion correction using FSL's eddy tool.

Volume censoring: DWI spatial resolution of DWIs was upsampled using cubic interpolation to a voxel size of 1.3mm3 to improve anatomical contrast and downstream template building, registration and statistics. Intensity normalisation was then performed across participants to increase anatomic delineation and improve statistics.

## Statistical modeling & inference

Model type and settings: We used a GLMM with average HADS-depression score derived from baseline and follow-up visit scores, as the independent variable. Subnuclei volumes and mean FC values were the dependent variable in these models which also had time between visits, age, intracranial volume and gender as co-variates, and participant as a random effect.

Effect(s) tested: Association between depression scores and both thalamic subnuclei FC and volume, using a GLMM.

Specify type of analysis: ☐ Whole brain ☐ ROI-based ☒ Both

Anatomical location(s): Thalamus

Statistic type for inference (See Eklund et al. 2016): Fixel based

Correction: FDR

## Models & analysis

| n/a | Involved in the study |
|---|---|
| ☒ | ☐ Functional and/or effective connectivity |
| ☒ | ☐ Graph analysis |
| ☒ | ☐ Multivariate modeling or predictive analysis |
